# Mutual Preservation: A Review of Interactions Between Cervicovaginal Mucus and Microbiota

**DOI:** 10.3389/fcimb.2021.676114

**Published:** 2021-07-13

**Authors:** Stylianos Vagios, Caroline M. Mitchell

**Affiliations:** Department of Obstetrics & Gynecology, Massachusetts General Hospital, Vincent Center for Reproductive Biology, Massachusetts General Hospital Research Institute, Boston, MA, United States

**Keywords:** cervicovaginal mucus, mucins, microbiome, female genital tract, reproductive health

## Abstract

At mucosal surfaces throughout the body mucus and mucins regulate interactions between epithelia and both commensal and pathogenic bacteria. Although the microbes in the female genital tract have been linked to multiple reproductive health outcomes, the role of cervicovaginal mucus in regulating genital tract microbes is largely unexplored. Mucus-microbe interactions could support the predominance of specific bacterial species and, conversely, commensal bacteria can influence mucus properties and its influence on reproductive health. Herein, we discuss the current evidence for both synergistic and antagonistic interactions between cervicovaginal mucus and the female genital tract microbiome, and how an improved understanding of these relationships could significantly improve women’s health.

## Introduction

Mucus, a gel-like substance composed of mucins, glycans, proteins, cholesterol and water, coats the lumen of multiple organs and plays a key role in regulating interactions between microbiota and epithelial surfaces (Varki et al., 2015). In the lower female genital tract (FGT), endocervical mucus protects the mucosal surface and helps prevent infection through entrapment of pathogens. Mucus in the FGT is distinct from other sites in its cyclic variation with changes in reproductive hormones. There are clear links between properties of cervical mucus and reproductive health outcomes, however, our understanding of specific pathways by which mucus characteristics influence reproductive health remains limited (Curlin and Bursac, 2013; Nunn et al., 2015; Smith-Dupont et al., 2017; Fernandez-Hermida et al., 2018; Hoang et al., 2020; Lacroix et al., 2020; Najmabadi et al., 2021).

Mucus properties such as consistency and protein composition can affect the intestinal bacterial community. Gut microbiota use exposed mucin glycans as both sites of attachment and as sources of nutrition. Commensal bacteria can degrade mucus by the production of proteases and/or sialidases, can stimulate mucin secretion directly or through increased gene transcription mediated by Toll-like receptor family members and alter mucus properties through microbial-host interactions (Smirnova et al., 2003; Radtke et al., 2012; Johansson and Hansson, 2016). While interactions between gut bacteria and intestinal mucus have been well described, patterns in the FGT are less clear.

Molecular classification of the microbiota has broadened our understanding of commensal bacteria in the FGT. In contrast to the gut, lower diversity of the microbial population in the vagina is associated with better reproductive health outcomes, such as lower incidence of bacterial vaginosis (BV), preterm birth and human immunodeficiency virus (HIV) acquisition (Gajer et al., 2012; DiGiulio et al., 2015; Gosmann et al., 2017). The vaginal microbiota is unique among body sites in that communities most associated with better clinical outcomes are low diversity, dominated by a single genus – *Lactobacillus* (Ravel et al., 2011; Anahtar et al., 2018). Sex hormones appear to have an important influence on composition of the vaginal microbiota (Johnson et al., 1985; Hillier and Lau, 1997; Eschenbach et al., 2000; Farage and Maibach, 2006; Bezirtzoglou et al., 2008), and also regulate qualities of cervicovaginal mucus (**Table 1**) (Chappell et al., 2014). The vaginal microbial community changes with hormonal transitions such as menarche, menses, pregnancy, and menopause (Zhou et al., 2007; Gajer et al., 2012; Hickey et al., 2015; Gliniewicz et al., 2019).

**Table 1:** Changes in cervicovaginal mucus properties and microbiome across menstrual phases and significant hormonal changes with menopause or pregnancy.

	Not Pregnant				Pregnant	References
	Follicular phase	Ovulatory phase	Luteal phase	Menopause		
Mucus Type	G-	S, L	G+	n/a	G+** (Gp)	(Odeblad, 1983; Odeblad, 1997)
Amount	↓	↑	↓	↓	↑	(Reynoso-Prieto et al., 2019)
Viscosity	+	–	++	+	++	(Odeblad, 1983; Odeblad, 1997)
Water content	Low	High	Low	Low	Low	(Curlin and Bursac, 2013; Reynoso-Prieto et al., 2019)
Mucin concentration						
MUC1	High	High	Low	Low	n/a	(Huggins and Preti, 1981; Moncla et al., 2016)
* MUC4*	Low	High	Low	Low	
* MUC5B*	Low	High	Low	Low	
* MUC5AC*	No dif.	No dif.	No dif.	No dif.	
* MUC7*	No dif.	No dif.	No dif.		
Glycan concentration	+	++	–	n/a	n/a	(Andersch-Bjorkman et al., 2007)
Oligosaccharides acidic profile	More acidic	More neutral	More acidic	n/a	n/a	(Argueso et al., 2002; Andersch-Bjorkman et al., 2007)
Total protein	+	–	+	n/a	n/a	(Reynoso-Prieto et al., 2019)
Lactoferrin	+++	+	++	–	–	(Mitsukawa et al., 2006; Keller et al., 2007; Becher et al., 2009; Wira et al., 2011)
Immunoglobulins	+	–	+	n/a	++	(Schumacher et al., 1977)
Lysozyme	+	–	+	–	–	(Schumacher et al., 1977)
Defensins	+++	+	++	–	+	(Keller et al., 2007; Xu et al., 2008; Wira et al., 2011)
Secretory leukocyte protease inhibitor (SLPI)	+++	+	++	+	++	(Shimoya et al., 2006; Keller et al., 2007; Wira et al., 2011)
Microbiome	Progressive increase in *Lactobacillus* spp. concentration Progressive decrease in non-*Lactobacillus* spp. concentration			Decreased concentration of *Lactobacillus* and BV-associated bacteria	Less rich, less diverse, increase in *Lactobacillus* concentration	(Hillier and Lau, 1997; Eschenbach et al., 2000; Gupta et al., 2020)

↑ = increased; ↓ = decreased; - = no change; number of + indicates relative concentration in vaginal fluid; n/a = no data available.

The contribution of cervicovaginal mucus to the composition of the FGT microbiome is unknown. Here we review what is known of the impact of cervicovaginal mucus on vaginal microbiota and vice versa. We discuss FGT mucus structure, how mucin properties regulate microbiota and how “optimal” and “non-optimal” bacterial populations modulate CVM. The purpose of this review is to provide an updated overview of the current knowledge, identify gaps, and suggest future directions for research.

## Cervicovaginal Mucus Composition

Cervical mucus is produced by epithelial cells within cervical crypts and is composed mainly of water, and a complex mixture of proteins, lipids, cholesterol, and inorganic ions. Mucins are glycoproteins that serve as a major structural component of mucus and are responsible for its viscous properties. Two types of mucins can be found in cervical mucus: secreted or gel-forming (MUC2, MUC5AC, MUC5B, and MUC6) and membrane-spanning (MUC1, MUC4, and MUC16) (Gipson et al., 1997; Gipson, 2001). The dominant gel-forming mucus is MUC5B and the major membrane-spanning mucus is MUC4 (Gipson et al., 1999; Gipson, 2001). The combination of cervical mucus secreted from the os and vaginal fluid (i.e. secretions from the Bartholin’s and Skene’s glands, plasma transudate, exfoliated cells, bacterial byproducts, bacteria and local immune cell secretions) is termed cervicovaginal mucus (CVM) (Huggins and Preti, 1981; Henderson et al., 2007; Srinivasan and Fredricks, 2008; Zegels et al., 2010). Vaginal epithelial cell MUC gene expression is lower than endocervical cells, suggesting that most mucins come from the cervix (Gipson et al., 1997).

Historically, gynecologists described four main types of cervical mucus (G-, G+, L, and S), all defined by the impact on fertility (**Table 1**) (Odeblad, 1983). At the beginning of luteal phase, viscous G- mucous has a substantial amount of white blood cells and acts as a barrier to semen ascension to the uterus. Increasing progesterone through the luteal phase supports a more viscous mucous type G+, containing more white blood cells, increasing the barrier to ascent of sperm. With rising estrogen in the follicular phase, type L contains fewer white blood cells, has medium viscosity, and allows more sperm motility. At peak estrogen around ovulation, type S has minimal viscosity and no white blood cells, allowing sperm to reach the uterus (Odeblad, 1983; Odeblad, 1997; Menarguez et al., 2003).

These historical descriptions primarily capture biologic variation in the consistency and amount of CVM. Biochemically, mucin secretion also varies within the menstrual cycle. An inverse correlation exists between serum progesterone levels and MUC5B mRNA expression (Gipson et al., 1999) while total cervical mucus and MUC5B secretion correlate positively with estrogen levels. Therefore, MUC5B is at its highest levels midcycle, and drops significantly in the luteal phase (Gipson et al., 2001). Expression of the transmembrane mucin MUC4 follows a similar pattern with a peak at midcycle and drop in the luteal phase (Gipson et al., 1999). The high midcycle water content of hydrophilic MUC5B may create a more patent endocervical canal, facilitating sperm motility and penetrance (Gipson et al., 2001). The glycosylation status of mucins also varies with the menstrual cycle, demonstrating increased carbohydrate concentration in secreted mucins and more neutral (*vs*. acidic) oligosaccharides at midcycle (Argueso et al., 2002; Andersch-Bjorkman et al., 2007).

## Impact of Cervicovaginal Mucus on Genital Microbiota

Mucus in the FGT serves both as physical and biochemical barrier against infectious pathogens. CVM contains immunoglobulins, antibacterial enzymes and antibacterial peptides, such as lysozyme, lactoferrin and defensins (Mitchell et al., 2015; Adnane et al., 2018; Elovitz et al., 2019). In mice, colonic epithelia mucus layers densely populated with MUC2 create a physical barrier that blocks bacteria-epithelium interaction (Johansson et al., 2008). Proteolytic cleavage of MUC2 loosens the structure of the outer intestinal mucus layer, allowing bacteria to colonize (Johansson et al., 2008). Moreover, mucus serves as a physical trap for particles (Bakshani et al., 2018). Cell-surface mucins can act as ligands for mucosal pathogens; the cell subsequently discards the extracellular mucus component to which pathogens are attached (Dhar and McAuley, 2019). In the FGT, CVM can trap organisms as small as herpes simplex virus (Schroeder et al., 2018). *In vitro*, mucins trap human immunodeficiency virus-1 (HIV-1) to a greater degree than similarly sized particles, suggesting charge-based or pathogen-specific effects beyond physical trapping (Mall et al., 2017).

**Figure 1:**
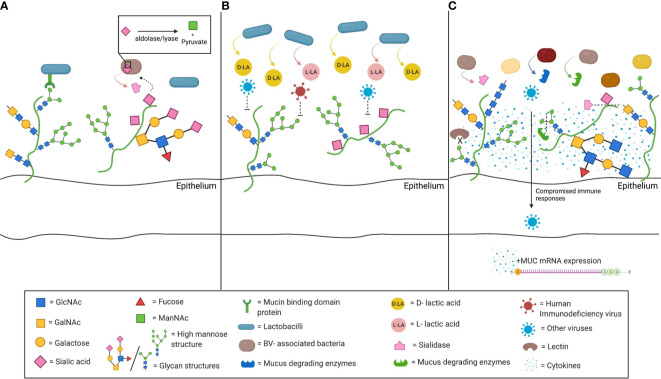
Schematic depiction of interactions between cervicovaginal mucus (CVM) and microbiota. **(A)** CVM may drive the composition of the commensal vaginal microbial community. Mucin binding protein (MucBP) domains and other binding factors allowing adhesion to mucins have been found on several lactobacilli, including the common vaginal species *L. crispatus* and *L. gasseri*. In addition, mucus constituents can be used as nutrient sources by commensal bacteria. Sialidase producing bacteria are able to catabolize the sialic acid present on mucins through an internal aldolase/lyase reaction. **(B)** Beneficial microbes, such as *L. crispatus*, also drive functional changes in the CVM. *L*. *crispatus* produces both D-lactic acid (D-LA) and L-LA, and CVM from women with *L. crispatus*-dominance has been associated with increased entrapment of viruses, including human immunodeficiency virus (HIV). **(C)** Bacterial vaginosis (BV)- associated bacteria can alter CVM properties. They may express sialidase and other mucus degrading enzymes, such as mucinases or other galactosidases. Sialidase production is associated with many of the adverse health outcomes related with BV, such as preterm birth, intrauterine infection during pregnancy, or invading infection. The lower sialic acid and the high mannose abundance, that have been correlated with BV-associated bacteria populations, along with the associated impaired lectin binding further compromise that innate immune responses in the lower genital tract. The resulting inflammatory environment has been shown to increased mucin mRNA expression and abundance (Figure created with BioRender.com).

Mucus may also drive the composition of the commensal vaginal microbial community (**Figure 1A**). Bacterial adhesion to mucins and glycans is proposed as a means of host positive selection of beneficial microbes (McLoughlin et al., 2016). The intestinal mucus layer serves as a natural habitat for “mucus-associated microorganisms” by serving as an attachment site (Paone and Cani, 2020). In the gastrointestinal tract, commensal lactobacilli have genes encoding mucin binding protein or other binding factors allowing adhesion to mucins. Similar genomic features are noted in *L. crispatus* and *L. gasseri*, two of the most common bacteria of the vaginal microbiota (Boekhorst et al., 2006; Velez et al., 2007; Van Tassell and Miller, 2011; Dudik et al., 2020). The ability to use CVM may facilitate *Lactobacillus* dominance of the vaginal microbiome.

Differences in glycosylation profile add additional intra-individual variability, and may be linked to traits such as blood type or secretor status (Thomsson et al., 2005). In the gut, secretor status and/or blood type is associated with differences in microbial colonization (Rausch et al., 2011; Davenport et al., 2016; Gampa et al., 2017; Kolde et al., 2018). Few studies examine the role of mucins in driving FGT microbial communities. Changes in mucin composition due to hormones, or disruption of CVM by pathogen mucinase activity could limit *Lactobacillus* binding, and “non-optimal” microbial populations could prevail (Dudik et al., 2020). In the gut, different mucin glycosylation patterns are instrumental to microbial tropism (Tailford et al., 2015). *In vitro* experiments demonstrate that *H. pylori* rarely grows in a mucin environment rich in a 1,4-GlcNAc-capped O-glycans (Kobayashi et al., 2009) and that transaldolase is an important factor promoting *Bifidobacterium bifidum* colonization (Gonzalez-Rodriguez et al., 2012).

Nutrient availability in cervicovaginal mucus could also drive shifts in community. Carbon sources such as glycogen are associated with *Lactobacillus*-dominant microbial populations (Mirmonsef et al., 2014). However, most lactobacilli present in the lower FGT do not directly metabolize glycogen, rather relying on glycogen degradation by host α-amylase (Spear et al., 2014; Nasioudis et al., 2015). There are some amylase-producing species of lactobacilli (e.g. *L. fermentum*) (Padmavathi et al., 2018). *L. crispatus* strains with a putative pullalanase type I gene can grow with glycogen as a carbon source, suggesting some strains may be able to directly utilize this sugar (van Der Veer et al., 2019). There is significant intraindividual variability in FGT glycogen concentrations and prolonged low glycogen states such as those seen in postmenopausal women favor microbial populations not dominated by lactobacilli (Mirmonsef et al., 2014; Mirmonsef et al., 2015). Similarly, mucins could also serve as a nutrient source for a number of bacteria. Human-derived mucins from different intestinal sites revealed over one hundred different oligosaccharides, which intestinal microbiota use as an energy source. The variety of carbon sources in mucins could contribute to different patterns of bacterial colonization along the gastrointestinal tract (Robbe et al., 2004). Several microorganisms found commensally in the vagina produce enzymes like glycosidases and proteinases, allowing them to degrade mucins and consume their glycans (Lewis et al., 2013; Werlang et al., 2019). Sialidase positive bacteria, such as *Gardnerella vaginalis* or *Prevotella bivia*, are able to catabolize sialic acid in the cervicovaginal mucins, releasing Neu5Ac (Smayevsky et al., 2001; Santiago et al., 2012; Lewis et al., 2013; Gilbert et al., 2019; Agarwal et al., 2020). They transport Neu5Ac intracellularly and catabolize it by an aldolase/lyase reaction (Lewis et al., 2013) which may confer a survival benefit in some nutrient deprived situations (Olmsted et al., 2003). Synergy between pathogenic species is also demonstrated by glycan cross-feeding supporting the concurrent growth of BV-associated species *G. vaginalis* and *Fusobacterium nucleatum*, a pathogen linked to intrauterine infection (Agarwal et al., 2020).

Mucins can regulate microbial gene expression, which may influence microbial colonization and function. MUC5AC, MUC2 and MUC5B exhibit potent inhibition of *Candida albicans*, a common pathogen of the FGT, downregulating virulence genes and inhibiting formation of hyphae (Kavanaugh et al., 2014). These same mucins downregulate virulence genes in the respiratory pathogen *P. aeruginosa* and decrease biofilm formation (Wheeler et al., 2019). Pre-incubation of *L. reuteri* (a common gut species) with gastric mucin led to greater adherence to HT-29 colon epithelial cells, and upregulation of bacterial surface adhesion proteins (Dudik et al., 2020). Apart from *C. albicans*, few genital tract microbes have been evaluated in these types of experiments.

Commensal bacteria co-exist in a fine balance with host innate immune responses. In addition to physical and nutrient properties, antimicrobial factors found in mucus may play a role in ensuring peaceful equilibrium. In the gut, regenerating family member 3 (REG3) protein (an antibacterial lectin) creates a zone of limited contact between the microbiota and the epithelium, ensuring immune quiescence. Commensal bacteria induce greater immune responses in REG3-gamma deficient mice (Vaishnava et al., 2011). In the respiratory tract, MUC1 can act as a negative regulator of inflammation induced through toll-like receptor (TLR) pathways (Dhar and McAuley, 2019). In the cervix, several antimicrobial peptides are found in mucus (e.g. secretory leukocyte protease inhibitor -SLPI-, lactoferrin, cathelicidin, defensins). SLPI, a potent inhibitor of leukocyte elastase, cathepsin G, and trypsin, is produced at the endocervical epithelium and is present in the cervical tissue and mucus (Moriyama et al., 1999). Epithelial and immune cells contribute to the production of other antimicrobial peptides found in the CVM such as β-defensins, elafin, calprotectin, cathelicidin, lactoferrin, α-defensins and lysozyme (Hein et al., 2002; Valore et al., 2002; Klotman and Chang, 2006; Keller et al., 2007; Levinson et al., 2009; Ghosh et al., 2010; Shust et al., 2010; Wira et al., 2011; Yarbrough et al., 2015). Regulation of these peptides in the FGT with hormonal cycles has been described (**Table 1**), but their integration with mucins, geographic orientation in the mucus layer and interaction with microbes at the mucosal surface have not been evaluated.

Focused research on whether and how CVM mucins and glycans influence vaginal colonization by particular microbiota, and how they modulate host-microbe interactions will likely offer new perspectives on how to promote optimal vaginal health.

## Influence of “Optimal” and “Non-Optimal” Microbiota on Cervicovaginal Mucus

In the gut, the commensal bacteria composition induces changes in intestinal mucus (Schroeder, 2019). In mice, gut microbiota promote production of proper mucus by affecting goblet cell numbers (Schroeder, 2019). In addition, the microbiota activate an enzyme, meprin β protease, necessary for the release of mucous from goblet cells (Johansson et al., 2015). The gut microbiota also influence mucin glycosylation profiles and local mucus thickness (Li et al., 2015; Corfield, 2018; Paone and Cani, 2020). In the FGT, sex hormone levels clearly impact mucus properties, and have been associated with changes in microbiota (**Table 1**). However, the impact of the local microbial community on CVM is incompletely described (Nunn et al., 2015).

### Interaction of “Non-Optimal” Cervicovaginal Microbiota With Mucins

Several bacteria associated with BV have the ability to degrade mucus (Briselden et al., 1992; Howe et al., 1999; Ravel et al., 2011). BV, a common cause of vaginal discharge, is characterized by a polymicrobial imbalance in favor of anaerobic bacteria, and is associated with increased rates of sexually transmitted infections (STIs), pelvic inflammatory disease (PID), and unfavorable obstetric outcomes (Hillier et al., 1988; Hillier et al., 1995; Svare et al., 2006; Brotman et al., 2010; Cohen et al., 2012). Sialic acid (SA) is a significant mucin glycan; the specific SA residue and attached sugars are crucial determinants of its function (Schauer, 2009). Production of the SA-degrading enzyme, sialidase, has been detected in a number of strains of *Gardnerella, Prevotella, Bacteroides*, and other BV-associated bacterial species (**Figure 1A**) (Briselden et al., 1992; Smayevsky et al., 2001; Santiago et al., 2011; Lewis et al., 2013). Lower SA concentrations are detected in women with BV (Smayevsky et al., 2001). Degradation of mucus by sialidase may contribute the watery discharge seen in BV, while elimination of the cervical mucus barrier may facilitate upper genital tract infection (Santiago et al., 2011).

The removal of SA residues renders mucins vulnerable to further degradation by proteases secreted by BV-associated bacteria and other pathogens such as *Trichomonas vaginalis* (Lehker and Sweeney, 1999; Kairys and Garg, 2020). BV-associated bacterial species secrete a variety of other mucus degrading enzymes, such as mucinases, sulfatases, galactosidases, and prolidases. These likely also impact the mucus barrier, though are less thoroughly studied (Howe et al., 1999; Cauci et al., 2005; Pleckaityte et al., 2012; Moncla et al., 2016). Alterations in physical and biochemical properties of mucus have been linked to preterm birth and intrauterine infection (Critchfield et al., 2013; Smith-Dupont et al., 2017), as have alterations in microbiota (DiGiulio et al., 2015). Sialidase levels, likely driven by BV-associated microbes, independently correlate with these obstetric complications (McGregor et al., 1994; Cauci and Culhane, 2011). Additional mucus alterations seen in women with BV, such as lower high mannose glycans, suggest other pathways through which bacterial communities may increase susceptibility to infections (Moncla et al., 2016).

Furthermore, the bacterial community in the FGT can induce production of mucus and mucins (**Figure 1C**). Women with BV have higher levels of MUC1, MUC4, MUC5AC, MUC5B and MUC7 than women without BV (Borgdorff et al., 2016; Moncla et al., 2016). This may be due to direct stimulation by BV-associated organisms and their byproducts, (Dohrman et al., 1998; Radtke et al., 2012) or BV-associated inflammation triggering mRNA upregulation (Li et al., 2003; Smirnova et al., 2003). Alternatively, increased mucin production may be a reaction to degradation by enzymes secreted by BV-associated microbes.

### Effect of “Optimal” Cervicovaginal Microbiota on Cervicovaginal Mucus Function

Beneficial microbes drive functional changes in mucus. CVM from women with *L. crispatus*-dominated vaginal microbiota prevents *in vitro* HIV infection better than CVM from women with diverse microbial communities (**Figure 1B**) (Lai et al., 2009). This enhanced protection appears to be due in part to the presence of metabolites from the lactobacilli, specifically D-lactic acid (D-LA), produced by *L. crispatus* but not *L. iners* (Boskey et al., 2001; Witkin et al., 2013; Nunn et al., 2015). The acidity supports hydrogen bonding between the viral surface and mucin carboxyl groups, though the reason for the difference in efficacy between the D- and L- isomers is not clear.

## Future Research Directions

In contrast to the gastrointestinal tract (Etienne-Mesmin et al., 2019), the FGT has limited experimental models in which to study mucin – microbiota interactions (Herbst-Kralovetz et al., 2016). One commonly used immortalized endocervical epithelial cell line is generally considered not to produce mucus (End1) while another does (A2EN) (Radtke et al., 2012). Collection of cervical mucus and assessment of physical and biochemical properties *in vitro*, or co-culture of microbes and mucus or mucins have provided additional insight (Muscariello et al., 2020). The source and processing of mucins for use in these experiments is important to retain their *in vivo* biologic activity (Kavanaugh et al., 2014; Wheeler et al., 2019). It is hoped that novel “organ-on-a-chip” technology can develop more comprehensive and high-fidelity models for study of mucin-microbe interactions in a more holistic manner (Ingber, 2021).

## Conclusion

Mucus in the FGT has a clear association with vaginal microbiota, reproductive health outcomes and mucosal immune responses but is the least well understood part of that triumvirate. Cervicovaginal mucus is easily accessible, but model systems to facilitate research in this field are not widely available. In the gastrointestinal tract animal models have contributed significantly to our appreciation of interactions between mucus, commensal bacteria, and host immune responses. It seems likely that mucus and mucins play a similar role in the FGT: regulating and modulating interactions between host and microbiota through availability of binding sites or nutrients and modulating physical host-microbe interactions. Conversely, pathogenic bacteria may change FGT mucus properties, undermining its role in preventing infection or inflammation, leading to adverse gynecologic and obstetric outcomes. A better understanding of interactions between CVM and cervicovaginal microbiota could help explain the pathophysiology behind numerous gynecologic and obstetric outcomes, and significantly improve women’s health.

## Author Contributions

SV and CM drafted and revised the manuscript. All authors contributed to the article and approved the submitted version.

## Funding

This work was supported in part by a Harvard Catalyst Microbiome Pilot award and the Vincent Memorial Research Funds. Neither funder participated in design or execution of the work.

## Conflict of Interest 

CM receives grant funding from Merck, and has served as a consultant for Scynexis Inc.

The remaining author declares that the research was conducted in the absence of any commercial or financial relationships that could be construed as a potential conflict of interest.
